# Defect Passivation through (α-Methylguanido)acetic Acid in Perovskite Solar Cell for High Operational Stability

**DOI:** 10.1021/acsami.2c00231

**Published:** 2022-04-27

**Authors:** Guan-Woo Kim, Jihyun Min, Taiho Park, Annamaria Petrozza

**Affiliations:** ‡ Center for Nano Science and Technology@Polimi, Istituto Italiano di Tecnologia, Via Giovanni Pascoli 70/3, Milano 20133, Italy; § Department of Chemical Engineering, Pohang University of Science and Technology (POSTECH), 77 Cheongam-Ro, Nam-gu, Pohang 37673, Kyoungbuk, Korea

**Keywords:** perovskite, solar cell, 2D perovskite, passivation methods, stability

## Abstract

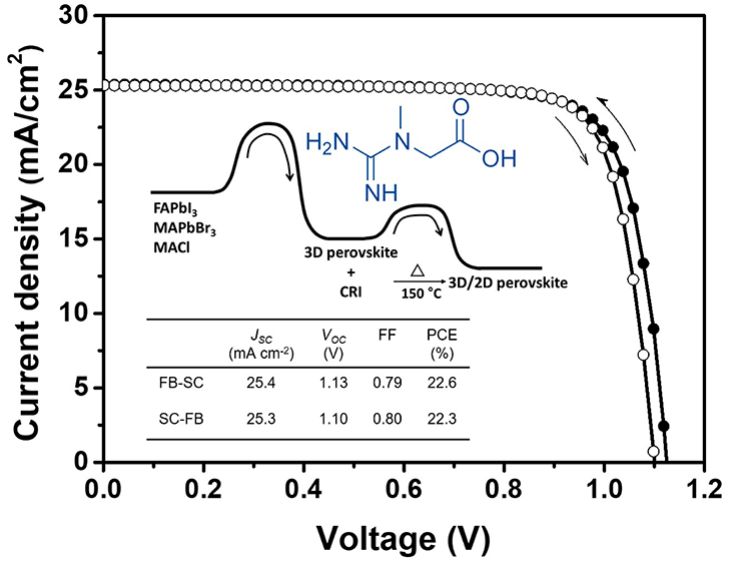

Defect passivation has become essential in improving efficiency and stability in perovskite solar cells. Here, we report the use of (α-methylguanido)acetic acid, also known as creatine, as a passivation molecule. It is employed both as an additive and as a surface passivation layer of perovskite thin films, given its multiple functional groups, which could address different defect sites, and its size, which could inherently affect the material structure. We prove that the surface passivation is more efficiently working by removing vulnerable defects on the surface. Hole and electron defect densities were reduced, leading to the highest power conversion efficiency of 22.6%. In addition, it can effectively protect the perovskite thin film and improve the operational stabilities in high thermal (85 °C) and humid conditions (50% relative humidity), suggesting a strong stability of the surface passivation layer.

## Introduction

Metal halide perovskites with ABX_3_ structure (A = cations, B = metal ions, X = halide), where A cations are surrounded by halide corner-sharing octahedra of BX_6_ in the position of cuboctahedra, have shown enormous potential in the photovoltaic area, recently reaching 25.7% power conversion efficiency.^1−3^ In addition, depending on the composition of perovskites, they exhibited superior and peculiar properties, broadening their applications.^4−6^ Metal halide perovskites can be processed by solvent-mediated methods at temperatures close to room temperatures, unlike conventional methods for silicon or thin-film-based solar cells. This brings about a much higher density of defects (∼1 × 10^16^ cm^–3^) in perovskites than those in conventional semiconductors (∼1 × 10^14^ cm^–3^).^7,8^ Nevertheless, they do not seem to heavily affect the primary figures of merit of perovskite-based devices, which exhibit competitive efficiency. Thus, perovskites are called “defect tolerant” semiconductors, as confirmed by many researchers. This enables current achievement.

The other side of perovskites is the instability. Although most defects are inactive in terms of efficiencies, they act as vulnerable sites for external stimuli such as moisture, oxygen, and heat by providing degradation pathways, largely contributing to intrinsic instabilities.^9−12^ Some halide defects promote ion migration in the perovskite layer and form phase-segregated regions.^9^ In addition, trapped charges in such defects critically accelerate the degradation of the perovskite layer.^10^ In CH_3_NH_3_PbI_3_, they facilitate the formation of volatile CH_3_NH_2_ under humid conditions, leaving inert PbI_2_. They can also promote the formation of superoxide (O_2_^–^) by providing additional electrons to oxygen in air under illumination, which destroys the structure of the perovskite layer.^11^ Especially, when charge carriers are trapped in halide interstitials, metastable state (I0) forms and it leads to I_2_ molecules by collision.^12^ They will move toward the surface and grain boundary, finally reacting with undercoordinated lead sites or leaving the perovskite. As it forms new kinds of defects and accelerates degradation, in this aspect, perovskite is not “defect tolerant”. In other words, the performance of perovskite solar cell (PSC) can be partially defect-tolerant, whereas the stability is clearly defect-intolerant.^13^ There have been many studies to find effective passivating materials and to understand how they operate.^14−17^ There are two main approaches: one is to use additives by mixing in precursor or antisolvent solutions, which can affect perovskite growth from the initial stages; the other is to use a passivation layer on the perovskite layer, which can selectively function on the vulnerable surface defects. In this case, it also works as a protecting layer. A hydrophobic layer is generally favored to prevent the penetration of moisture, whereas a hydrophilic layer is sometimes employed as a sacrificial layer that absorbs moisture instead of the inner perovskite layer.^18−20^ Both methods to passivate defects are considered to be very effective, but the latter can be more intuitive and systematic because it does not interfere with the bulk perovskite growth. Because of the ionic character of perovskite, defects in PSCs can be deactivated using electrostatic interaction such as ionic or coordinate bonding, in contrast to the earlier generation of solar cells, which generally employed covalent bonding.^21−23^ For example, it is reported that theophylline, where N–H and C=O groups are located in an optimal configuration in the molecule, can effectively passivate Pb-related defects, achieving large improvement in both efficiency and stability.^24^ Another approach is to react with other materials to convert defects to other states for improving charge transfer and reducing charge recombination, which is similar to that employed in silicon solar cells.^25^ Especially, the formation of 2D perovskite is one of the generally employed methods. It can improve stability by converting energetically unstable defects and 3D perovskites on the surface into 2D layered perovskites, generally described as A′_2_A_*n*–1_B_*n*_X_3*n*+1_, where A′ is a long alkyl chain-containing cation. Longer alkyl chains in 2D perovskite guarantee better moisture stability. In addition, A requires more energy to leave the layered structure than A (CH_3_NH_3_^+^, MA^+^), contributing to intrinsically high structural stability.^26^ Typical and successful examples are phenylethylammonium iodide, *n*-butylammonium iodide, *n*-hexyl trimethylammonium bromide (HTABr), and 5-ammoniumvaleric acid iodide.^27−30^

Herein, we investigate the use of (α-methylguanido)acetic acid, which is called creatine.^31^ It is known to be involved in the process of energy production in animals. It is noted that for convenience, (α-methylguanido)acetic acid will be called creatine. It has functional groups (−COO^–^, −C(=NH)NH_2_) including oxygen and nitrogen, and thus it may interact with defect sites of different natures, whereas its size makes it a good candidate for the formation of 2D structures with improved structural stability by providing strong hydrogen bonding between molecules. We test it both as an additive during the thin film growth and a surface modifier. Creatine forms a 2D perovskite layer on the 3D perovskite layer when it is employed as a surface modifier. It efficiently removes the defects on the surface and acts as a passivation layer, whereas creatine just deactivates defect sites by providing electrostatic interaction when it is employed as an additive. As a result, PSCs employing (FAPbI_3_)_0.95_(MAPbBr_3_)_0.05_ exhibited the highest efficiency of 22.6% and improved stability even though it contains hydrophilic functional groups.

## Results and Discussion

For the application of creatine, its solubility was checked in common organic solvents. For isopropyl alcohol, dimethylformamide, dimethyl sulfoxide, and chlorobenzene, which are commonly used solvents in PSC processing, the solubility of creatine was too low (≤2 mg/mL) to see its effect. Hydroiodic acid (HI) was added to make creatine soluble by protonating nitrogen in the formamidine group. When 2 equiv. of HI were added, creatine showed high solubility (≥20 mg/mL) in all mentioned solvents (Figure S1). Strictly speaking, the product is different from creatine, and we call this CRI for convenience. We first investigated the ability of CRI as an additive in perovskite precursor. To see how it affects perovskite morphology, the surfaces of the perovskite layer with the addition of different concentrations of CRI were observed using a scanning electron microscope (SEM) (Figure S2). All perovskite layers exhibited similar morphologies, which implied CRI would not directly affect the perovskite growth. The structural properties also exhibited no changes in grazing-incidence wide-angle X-ray scattering (GIWAXS) and X-ray diffraction (XRD) (Figures S3–S5). PSCs were fabricated in the configuration of FTO/SnO_2_/(FAPbI_3_)_0.95_(MAPbBr_3_)_0.05_/spiro-OMeTAD/Au. When the molar concentration of added CRI was 7 mM, the efficiency was the highest, i.e., 21.6%, with respect to the reference sample presenting a power conversion efficiency (PCE) of 20.4% (Figure S6 and Table S1). Although we have observed decreased defect densities and the following effect (Figures S7–S9), this approach was effective but not impressive, and empirically, batch-to-batch variation was rather high.

**Figure 1:**
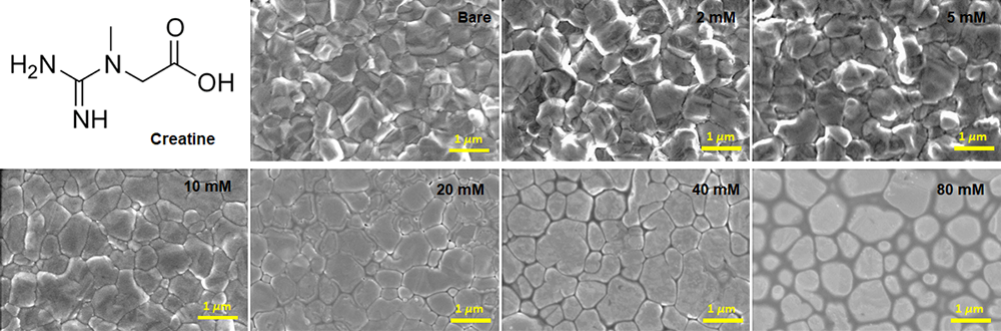
Chemical structure of creatine and top SEM images for perovskite morphology changes depending on the concentration of CRI solution.

CRI was then employed on the perovskite layer as a passivation layer. Contrary to the previous case, the morphology of the CRI-treated perovskite surface was remarkably changed (Figure 1). When compared to the SEM image of the bare perovskite layer, the perovskite grain became clear as the concentration of the CRI solution increased up to 10 mM. From the 20 mM CRI-treated perovskite layer, something was observed along the grain boundary and it became very obvious in the highly concentrated CRI-treated perovskite layer (40–80 mM). We assumed that it could be excessive CRI aggregates, which would have a harmful effect on photovoltaic performances because of their insulating property.

**Figure 2:**
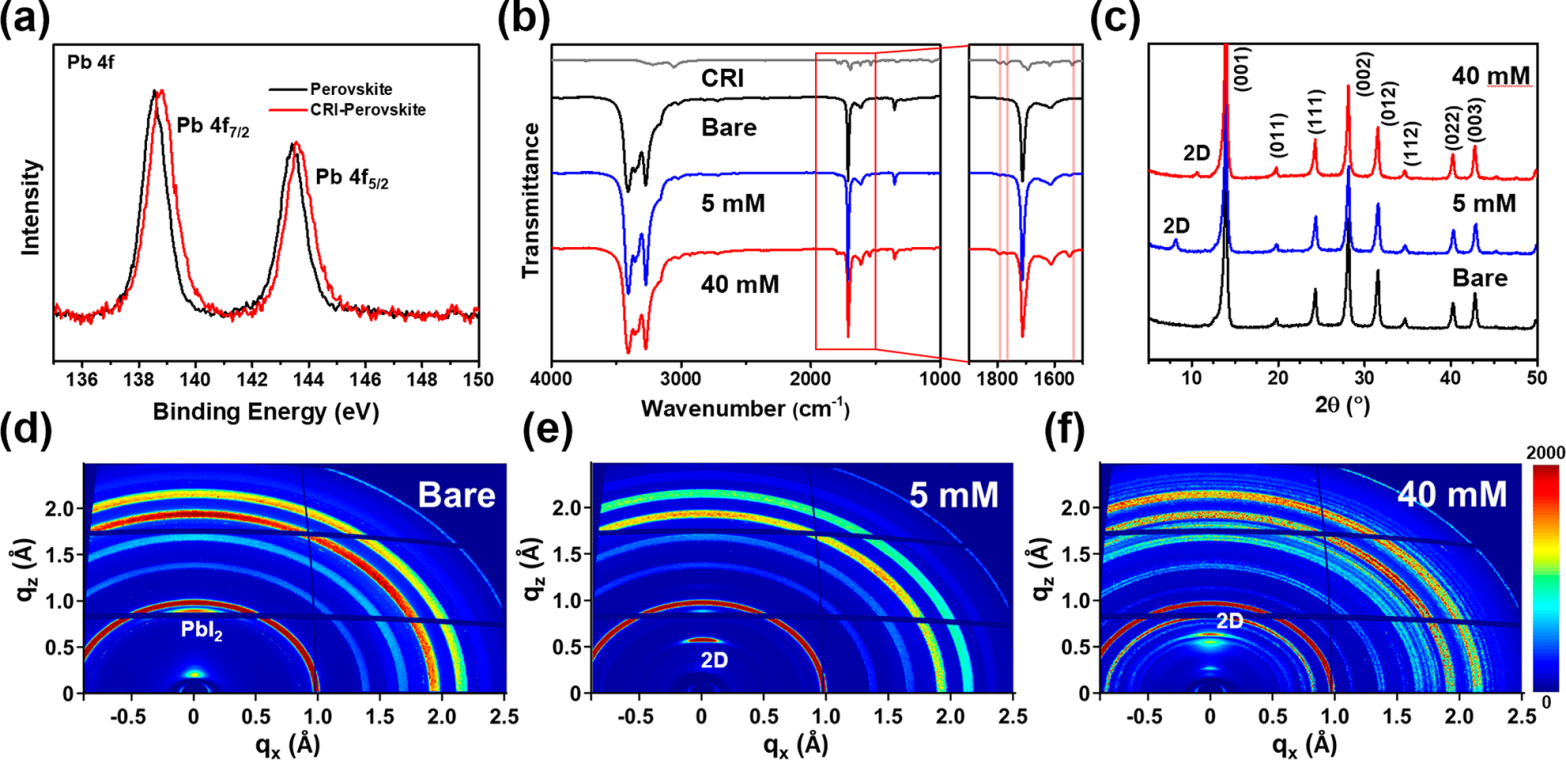
(a) XPS analysis for Pb 4f in CRI-treated perovskite sample. (b) FTIR results of CRI, bare perovskite and CRI-treated perovskite samples. (c) XRD results of bare perovskite and CRI-treated perovskite samples. GIWAXS of (d) bare perovskite, (e) 5 mM CRI-treated perovskite, and (f) 40 mM CRI-treated perovskite.

To further understand the role of CRI, we performed a series of analyses. In Figure 2a, we show the X-ray photoelectron spectroscopy (XPS) analysis of thin film surfaces. The CRI-treated perovskite layer exhibited two distinct peaks for Pb 4f 7/2 near 138.8 eV and Pb 4f 5/2 near 143.6 eV, slightly shifted toward higher binding energy compared to those of the bare perovskite layer (Pb 4f 7/2–138.5 eV, Pb 4f 5/2–143.4 eV). This indicates the existence of interaction between CRI and lead on the surface. The appearance of the O 1s characteristic peak (∼533.2 eV) in the CRI-treated perovskite layer indicates the successful introduction of CRI (Figure S10). Fourier-transform infrared (FTIR) spectroscopy also supports previous results (Figure 2b). In contrast to bare perovskite, C=O stretching peaks (1540, 1768, 1791 cm^–1^) appeared in CRI-treated perovskites. In addition, these peaks, which are indicated as hazy red lines, were slightly shifted, which can be a result of interaction between oxygen in the carboxyl group and lead. In XRD, several structural properties were observed different from the case that CRI was used as an additive (Figure 2c and Figures S11 and S12). As the concentration reached 5 mM, a 2D perovskite peak appeared at 8.14°. Then, as the concentration increased, another peak appeared at 10.6°, which can be estimated to be another 2D perovskite structured in closer stacking of organic layer, leading to smaller *d*-spacing. GIWAXS also supports the formation of a 2D perovskite layer (Figure 2d–f and Figures S13 and S14). In the bare sample, typical diffraction rings which stand for 3D perovskite were confirmed, indicating its randomly oriented polycrystalline structure (Figure 2d). In Figure 2e and f, additional peaks at *q*_z_ = 0.58 and 0.76 Å^–1^, which yield *d*-spacing values of 10.8 and 8.3 Å, are exactly matched with signals (8.1 and 10.6°) in XRD. 2D perovskite at *q*_z_ = 0.58 Å^–1^ has a well-aligned face on orientation, whereas the newly generated one is randomly oriented in the film, which can be not beneficial for device performances. It is noted that a high concentration of CRI can induce large disorder, generating a variety of randomly distributed structures. Accordingly, it is very important to choose the optimized concentration of CRI to promote the formation of intended 2D perovskite and to exclude the other.

**Figure 3:**
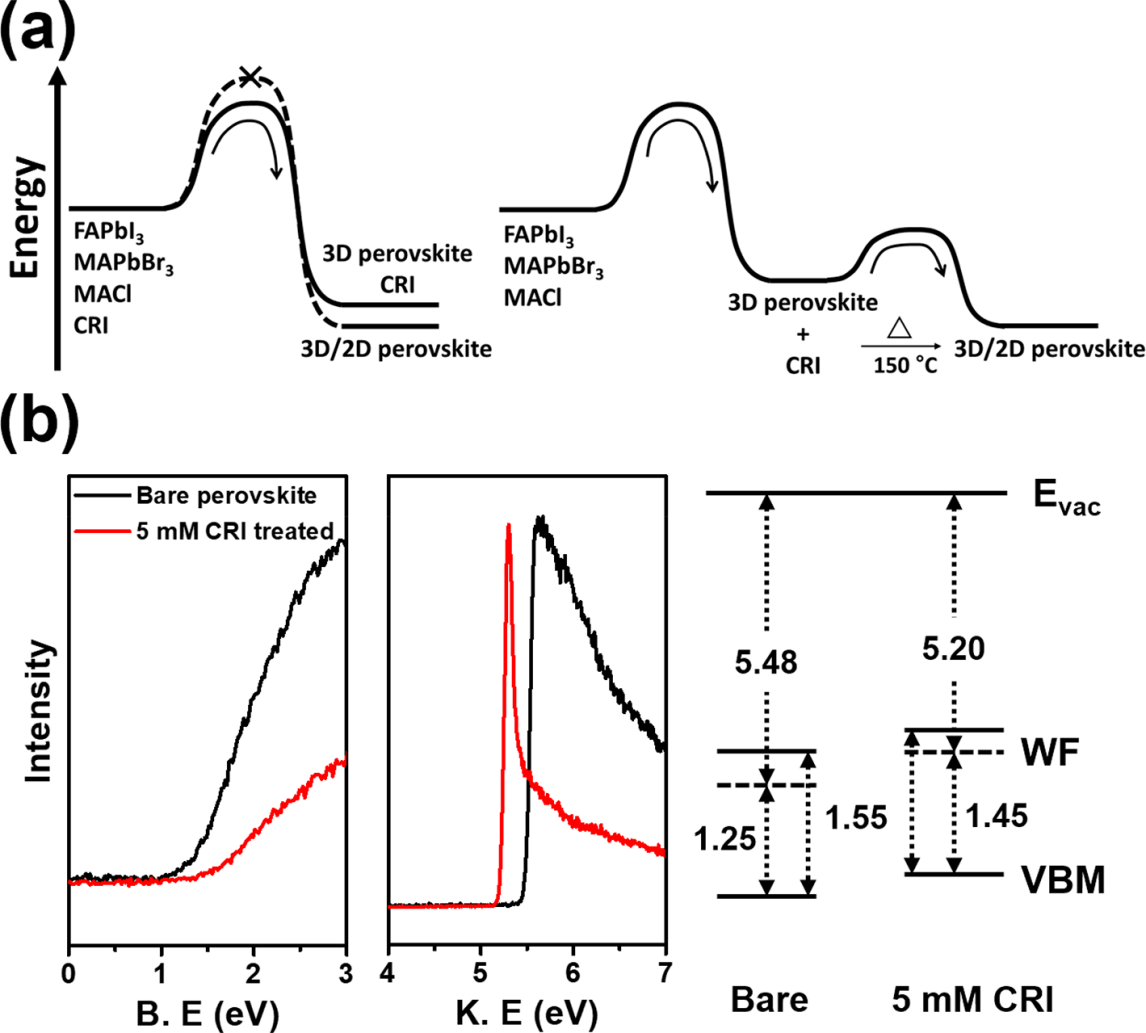
(a) CRI working mechanism as an additive in perovskite precursor and a passivation layer on the perovskite layer. (b) UPS measurement and calculated energy level alignment.

The working mechanism of CRI as an additive and a passivation layer can be demonstrated on the basis of previous observations (Figure 3a). When CRI is employed as an additive, it is dispersed in the precursor and is in direct competition with other organic cations for insertion into the perovskite lattice. However, it is more unfavorable than other small organic cations, leading to the higher activation energy for the reaction. As a result, CRI will be distributed near the lattice, stabilizing defect states by merely providing electrostatic interaction. On the other hand, the other case is to first form 3D perovskite. The thermal energy to overcome the smaller activation energy is then provided by annealing at 150 °C. It converts unstable defects and 3D perovskites on the surface into 2D perovskites, finally achieving a more stable 3D/2D structure. This structure not only provides the same electrostatic interaction but also removes defects states permanently, which is considered to be more advantageous. Before the fabrication of solar cells, we checked the energy level of CRI-treated perovskites using ultraviolet photoelectron spectroscopy. The conduction band maximum (CBM) and valence band maximum (VBM) are affected by surface modification. The CBM and VBM of the surface were raised, which is favorable for charge transfer between 3D perovskite (bulk) and hole transport materials (Figure 3b). When the concentration of the CRI solution was increased, the VBM of the perovskite on the surface was shifted below that of 3D perovskite (Figure S15). This can act as a barrier for smooth charge transfer, which is consistent with previous results.

**Figure 4:**
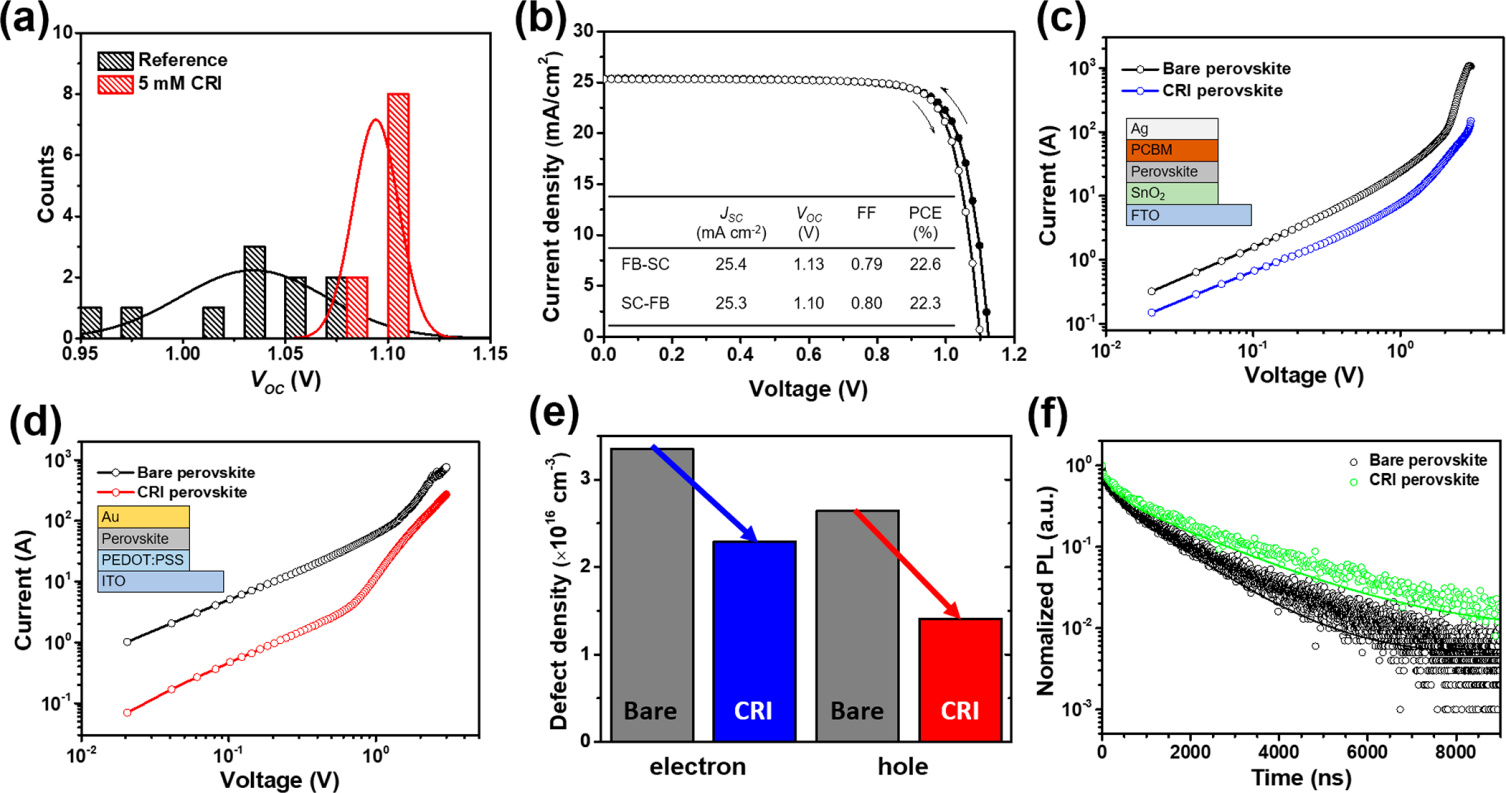
(a) *V*_OC_ histograms for reference and 5 mM CRI-treated PSCs. (b) *J*–*V* curves and photovoltaic parameters for CRI-treated champion cell. (c) SCLC measurements of electron-only devices. (d) SCLC measurements of hole-only devices. (e) Defect density changes after the introduction of a CRI passivation layer. (f) Transient PL measurements.

To find the optimal concentration of CRI solution, PSCs were fabricated in the configuration of FTO/SnO_2_/(FAPbI_3_)_0.95_(MAPbBr_3_)_0.05_/CRI passivation layer/spiro-OMeTAD/Au (Figure S16). At a 5 mM CRI solution concentration, the device exhibited the highest efficiency and reproducibility (Figure S17 and Table S2). When its concentration reached 5 mM, the device efficiency started to decrease, probably because of unintended 2D perovskite and excessive CRI aggregates, which impede the efficient charge transfer. The improvement was mainly attributed to an open circuit voltage (*V*_OC_) increase, which resulted from reduced nonradiative recombination through defect passivation (Figure 4a). The champion device exhibited the highest efficiency of 22.6% in reverse scan with negligible hysteresis in the planar device (Figure 4b), which is 10.8% higher than that of reference device (20.4%). External quantum yield (EQE) was measured to confirm the consistency with short circuit current (*J*_SC_) from the current density (*J*)–voltage (*V*) curve (Figure S18).

For further investigation on how CRI affects the defect density in the perovskite layer, we measured the space charge limited current (SCLC) and time-resolved PL (TRPL). Electron- and hole-only devices were fabricated to obtain defect densities from SCLC measurements. For electron defects, the defect density of the CRI-treated perovskite layer was 2.29 × 10^16^ cm^–3^, less than that of the bare perovskite layer, 3.35 × 10^16^ cm^–3^ (Figure 4c). In addition, the hole defect density was decreased from 2.64 × 10^16^ cm^–3^ to 1.41 × 10^16^ cm^–3^ (Figure 4d). Both results prove that CRI can passivate a variety of defects, reducing electron and hole defect densities (Figure 4e). When compared to defect densities of the perovskite layer using CRI additive, the effect of CRI was larger in using the CRI passivation layer, indicating a 31.64 and 46.59% reduction ratio of electron and hole defect densities, whereas the ratio was 12.16 and 24.91% for the CRI additive (Figure S7). This is attributed to CRI, as a passivation layer, being able to directly remove defect states as well as provide electrostatic interaction. TRPL was measured by changing the concentration of CRI. The lifetime tendency is exactly consistent with histograms of *J*–*V* parameters, indicating that the 5 mM CRI-treated perovskite layer exhibited the longest lifetime (Figure 4f and Figure S19), in good agreement with the reduced defect densities in the film. All of these results strongly support that a well-optimized CRI passivation layer effectively reduces the defect density in the perovskite layer, thereby improving the device performance.

**Figure 5:**
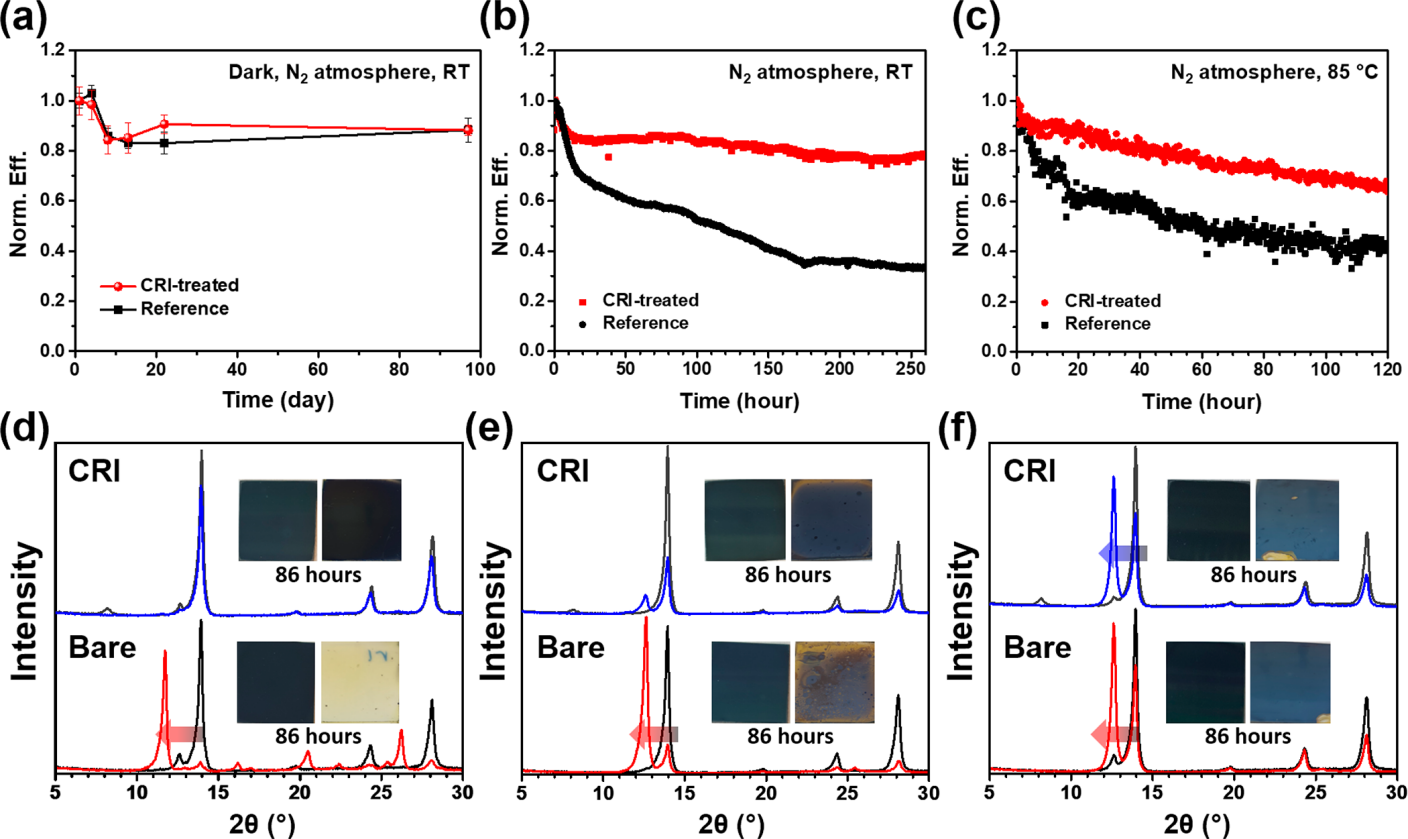
(a) Shelf life stability tests of PSCs. MPP tracking under a N_2_ atmosphere: (b) at room temperature (RT) and (c) at 85 °C. XRD changes after 86 h at such conditions (black line, 0 h; blue and red lines, after 86 h): (d) 50%RH, RT; (e) 50%RH, 85 °C; (f) > 80%RH, 85 °C.

Recent interest has focused on the stability of PSC. The stability of perovskite is much more improved in past years, but its intrinsically low stabilities against moisture, oxygen, and light are still the main concerns.^32−35^ Most degradation is accelerated by vulnerable defects that can trap the charges and provide the degradation pathway. The passivation of such defects can lead to a large improvement in stability as well as efficiency. First, we performed the shelf life stability test of PSC with or without CRI treatment (Figure 5a and Figure S20). The devices were stored in dark conditions under 20% relative humidity (RH). All devices maintained their initial efficiencies over 3 months, which indicates without any external stimulation, the stability of PSC no longer brings any issues. Instead, continuous illumination critically affects the stability of PSCs. Maximum power point (MPP) tracking was measured in different conditions under the N_2_ atmosphere. At room temperature, CRI-treated PSC maintained 78% of its initial efficiency over 250 h, whereas reference PSC exhibited rapid degradation, reaching 33% of its initial efficiency (Figure 5b). At the thermal condition of 85 °C, the tendency was exactly the same, that CRI-treated PSC maintained 66% of its initial efficiency over 120 h, whereas reference PSC maintained 42% (Figure 5c). Results in MPP tracking imply that defect passivation is very important because defects are deeply involved in ion migration and it is accelerated under continuous illumination conditions, thereby critically influencing device lifetime in real working conditions.

To further understand the material stability against various stimuli, we measured XRD upon time in various conditions. In ambient condition (∼50%RH), the representative peak of α phase perovskite (∼14°) disappears in the bare perovskite layer after 86 h, while a new peak around 11.8°, which stands for δ phase perovskite, appears (Figure 5d and S21). It means that α phase perovskite layer converts into δ phase perovskite layer, by losing photoactive properties due to its structural instability in the ambient condition. On the other hand, the CRI-treated perovskite layer well maintained its structure. When heating samples at 85 °C in ambient conditions, degradation of the α phase led to the formation of PbI_2_ (12.6°) in both samples (Figure 5e and Figure S22). It seems that external heating accelerates the release of volatile organic cations, which indicates a totally different degradation mechanism compared to the case in the ambient condition. The ratios of decrease in α phase peak and increase in δ phase or PbI_2_ peak were much smaller in CRI-treated perovskite layer. At last, samples were stored in highly thermal and humid condition (85 °C, over 80%RH). In this condition, both samples exhibited low stability, losing their gloss and getting faded (Figure 5f and Figure S23). This indicates that encapsulation is essential to resisting such a harsh condition. In these degradations, one of the common features is that the α phase peak of the CRI-treated perovskite layer starts to decrease after the 2D perovskite peak first disappears. This observation suggests that only when the 2D perovskite layer is degraded, the overall degradation of the solar cell begins. This can be correlated to CRI-treated PSCs exhibiting comparatively less rapid degradation at the initial stage than reference PSCs (Figure 5b, c).

**Figure 6:**
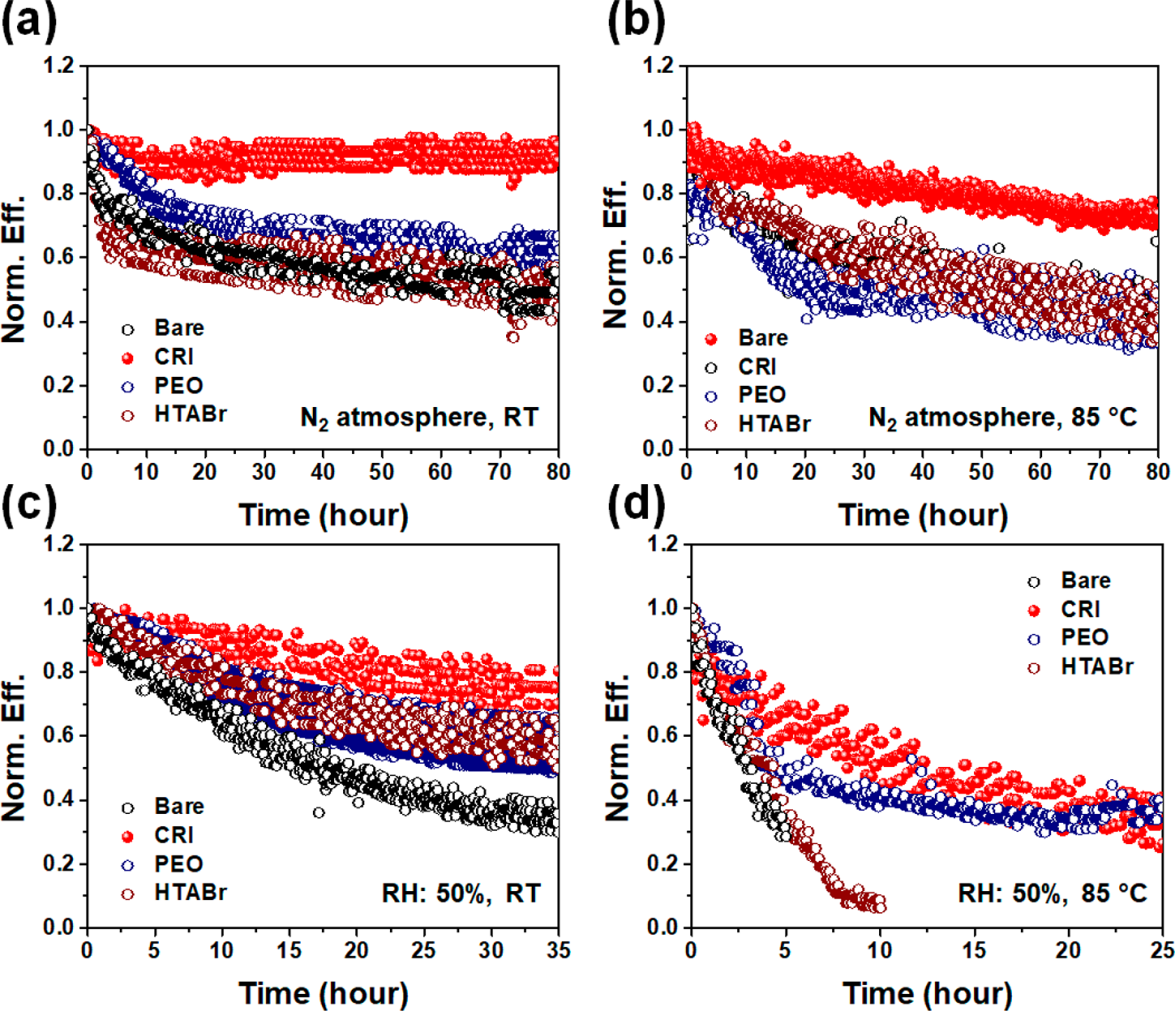
MPP tracking of four types of samples, bare perovskite, CRI-treated perovskite, PEO-treated perovskite, and HTABr-treated perovskite, in such conditions: (a) N_2_ atmosphere, RT; (b) N_2_ atmosphere, 85 °C; (c) 50%RH, RT; and (d) 50%RH, 85 °C.

For further evaluation of CRI passivation layer, we compared CRI with other representative passivation materials. We chose two different types of passivation materials: polyethylenoxide (PEO) and HTABr.^19,29^ As a passivation layer, PEO can provide a hydrophilic layer and HTABr can provide a hydrophobic layer with the formation of 2D perovskite layer. In the meantime, CRI can provide a hydrophilic layer with the formation of 2D perovskite layer. In the comparison between these representative materials, we can evaluate which types of materials can more efficiently contribute to stable and durable devices. Operational stability was monitored using MPP tracking in various conditions. Under a N_2_ atmosphere, CRI-treated PSC maintained its initial efficiency for 80 h, whereas others exhibited about 40% efficiency drop (Figure 6a). In high temperature condition (85 °C), CRI-treated PSC also exhibited the highest stability compared to others, maintaining 73% of its initial efficiency (Figure 6b). Same tests were conducted under ambient condition (50%RH). In this condition, the difference was more obvious. At the room temperature, CRI device maintained 70% of its initial efficiency for 35 h, whereas PEO and HTABr devices maintained 60% and the reference device maintained 40% (Figure 6c). Obviously, passivation layers lead to improved stability, among which the CRI layer was showing the best performances. At the 85 °C condition, the efficiency drops of samples were critically large. The resistivity of each device follows the following order: CRI, PEO, HTABr, and bare (Figure 6d). From these results, we can claim that a hydrophilic layer can efficiently protect a vulnerable perovskite surface and provide robustness as a few researchers have reported.^19,20^

## Conclusions

We employed CRI, modified from (α-methylguanido)acetic acid, as an additive in the perovskite layer and a passivation layer on the perovskite surface. CRI works more effectively when forming a 2D perovskite layer on top of the perovskite surface. Vulnerable defects are easily passivated by reaction with CRI, forming the well-aligned 2D perovskites upon annealing. Defect densities for hole and electron were decreased, thereby reducing nonradiative recombination. This resulted in high efficiency (22.6%) and stable devices. We confirmed that operational stabilities in thermal and humid conditions were significantly improved compared to bare perovskite devices and those passivated with the most commonly used surface passivation solutions. In comparison with other passivation materials, CRI, which can provide hydrophilic and 2D perovskite layers, was proved to lead to the most durable devices by sacrificing itself instead of the inner 3D perovskite layer.

## Experimental Section

### Materials

Lead iodide (PbI_2_, TCI), lead bromide (PbBr_2_, TCI), formamidinium iodide (FAI, Greatcell Solar Materials), methylammonium bromide (MABr, Greatcell Solar Materials), *n*-hexyl trimethylammonium bromide (HTABr, Greatcell Solar Materials), polyethylenoxide (PEO, Sigma-Aldrich), methylammonium chloride (MACl, Sigma-Aldrich), spiro-OMeTAD (Luminescence Technology corp.), FK209 (Luminescence Technology corp.), creatine (Sigma-Aldrich), hydriodic acid (Alfa Aesar), SnO_2_ colloidal solution (Alfa Aesar), dimethylformamide (DMF, Sigma-Aldrich), dimethyl sulfoxide (DMSO, Sigma-Aldrich), isopropyl alcohol (IPA, Sigma-Aldrich), chlorobenzene (CB, Sigma-Aldrich), diethyl ether (DEE, Sigma-Aldrich), acetone (Sigma-Aldrich), and ethanol (Sigma-Aldrich)

### Device Fabrication

FTO substrate was cleaned using detergent, deionized (DI) water, acetone, and isopropyl alcohol (IPA). The dried substrate was treated under UV-ozone for 15 min. Diluted SnO_2_ colloidal solution (5:1 vol %) was spin-coated with a speed of 3000 rpm for 30 s and then annealed at 150 °C for 30 min. Twenty milligrams per milliliter of creatine solution was prepared. To make it soluble, 2 equiv. of hydriodic acid was added. Finally, 150 mM of CRI solution (DMSO or IPA) was obtained. It was used by diluting in DMSO or IPA depending on the purpose of use (additive or passivation layer). Then, the prepared perovskite precursor was deposited on the substrate as reported (22.7% using dopant-free P3HT as a hole transport material).^29^ All depositions were processed in air (∼20% relative humidity). The spiro-OMeTAD solution was prepared in a composition of 72.3 mg of spiro-OMeTAD, 27.8 μL of tBP, 17.8 μL of Li-TFSI (520 mg mL^–1^ in AN), and 2 mg of FK209 in 1 mL of CB. It was then spin-coated at 5000 rpm for 30 s. Finally, a 100 nm thickness of gold electrode was thermally deposited.

### Device Characterization

The *J–V* curve and maximum power point efficiency (MPP) was obtained under simulated AM1.5 solar illumination using a class AAA solar simulator (Oriel Sol3A, Newport) and Keithley 2440. The illumination intensity was calibrated using a KG-5 filtered certified Si reference diode (area = 4 cm^2^, Newport) to be 100 mW cm^–2^. The *J–V* curves of all devices were measured by 0.2 V s^–1^ of scan rate with reverse (1.2 V to −0.2 V) or forward (−0.2 to 1.2 V) bias. The device active area is 0.0935 cm^2^ and Devices were measured in an ambient atmosphere at room temperature and 40–60% relative humidity. Operational stability was measured using CICCI Research products equipped with LED laser which can provide same spectrum with 1 Sun. It was measured under maximum power point.

### SEM Measurement

Field-emission scanning electron microscope (FE-SEM, Hitachi S 4800) was employed to obtain the optical top and cross-sectional images.

### PL Measurement

The TRPL and steady-state PL measurements were conducted using prepared samples: glass/perovskite + CRI or glass/perovskite/CRI layer. The samples were excited from the glass side under ambient conditions with excitation wavelength of 474 nm. Time-resolved photoluminescence (TRPL) was measured using time correlated single photon counting (TCSPC) system (HAMAMATSU/C11367–31). For TRPL measurements, a pulsed laser source was laser diode with a wavelength of 474 nm, a repetition rate of 100 kHz, fluence of ∼4 nJ/cm^2^, and a pulse width of 70 ps. Steady-state photoluminescence (PL) was measured using monochromator and hybrid photomultiplier detector (PMA Hybrid 40, PicoQuant GmbH).

### SCLC Measurement

The device structure of FTO/SnO_2_/perovskite/PCBM/Ag or ITO/PEDOT:PSS/perovskite/Au was prepared. Depending on the purpose of use, CRI was added to perovskite or deposited on the perovskite layer. Current–voltage curves are obtained from scanning from 0 to 5 V to evaluate the trap densities (*n*_t_) of devices following the equation . The film thickness is around 400 nm, and the dielectric constant comes from the literature.^36^

## Supporting Information Available

SEM images, GIWAXS, XRD, box charts, defect densities from SCLC curves, XPS, UPS, EQE, TRPL, and stability tests. (PDF)
