# Supplementary material for: Defect Passivation through (α-Methylguanido)acetic Acid in Perovskite Solar Cell for High Operational Stability
Source: ACS Appl Mater Interfaces. 2022 Apr 27;14(18):20848–55. doi: 10.1021/acsami.2c00231 (PMC9100480; doi:10.1021/acsami.2c00231)
Supplement: Supplementary file 1 — am2c00231_si_001.pdf [file am2c00231_si_001.pdf]

Supporting Information

Defect Passivation through (α-Methylguanido)acetic Acid in Perovskite Solar Cell for High Operational Stability

Guan-Woo Kim,‡[a] Jihyun Min,‡[b] Taiho Park*[b] and Annamaria Petrozza*[a]

a. Center for Nano Science and Technology@Polimi, Istituto Italiano di Tecnologia, Via Giovanni Pascoli 70/3, 20133 Milano, Italy

b. Department of Chemical Engineering, Pohang University of Science and Technology (POSTECH), 77 Cheongam-Ro, Nam-gu, Pohang, Kyoungbuk, Korea 37673

* Corresponding author

E-mail addresses: Annamaria.Petrozza@iit.it (A. Petrozza), taihopark@postech.ac.kr (T. Park)

‡ These authors contributed equally to this work.

Contents

Figure S1. Solubility of creatine in DMF, DMSO and IPA.

Figure S2. Top SEM images of perovskite layer with different concentration of CRI additives

Figure S3. GIWAXS of perovskite layer with different concentration of CRI additives

Figure S4. Radial cuts of perovskite layer with CRI additives.

Figure S5. XRD of perovskite layer with different concentration of CRI additives

Figure S6. Histograms of perovskite solar cells with different concentration of CRI additives

Figure S7. Current-voltage curves of SCLC devices and defect densities

Figure S8. XPS of Pb 4f and O 1s of perovskite layer with and without CRI additives

Figure S9. Steady state PL of perovskite layer with and without CRI additives prepared on bare glass.

Figure S10. XPS of O1s of perovskite with CRI passivation layer

Figure S11. XRD of CRI

Figure S12. XRD of perovskite layer with different concentration CRI passivation layer

Figure S13. GIWAXS of perovskite layer with different concentration CRI passivation layer

Figure S14. Radial cuts of perovskite layer with CRI overlayer.

Figure S15. UPS of perovskite layer with 40 mM CRI passivation layer

Figure S16. Cross sectional SEM image of planar perovskite solar cell

Figure S17. Box charts of devices using different concentration CRI passivation layer

Figure S18. EQE of champion device

Figure S19. TRPL and steady state PL of perovskite layer with different concentration CRI passivation layer

Figure S20. Shelf life stability tests: other parameters

Figure S21. XRD changes with time at 50 %RH, RT.

Figure S22. XRD changes with time at 50 %RH, 85 °C.

Figure S23. XRD changes with time at >80 %RH, 85 °C.

Table S1. Photovoltaic parameters of PSC employing CRI additives.

Table S2. Photovoltaic parameters of PSC employing CRI passivation layer.

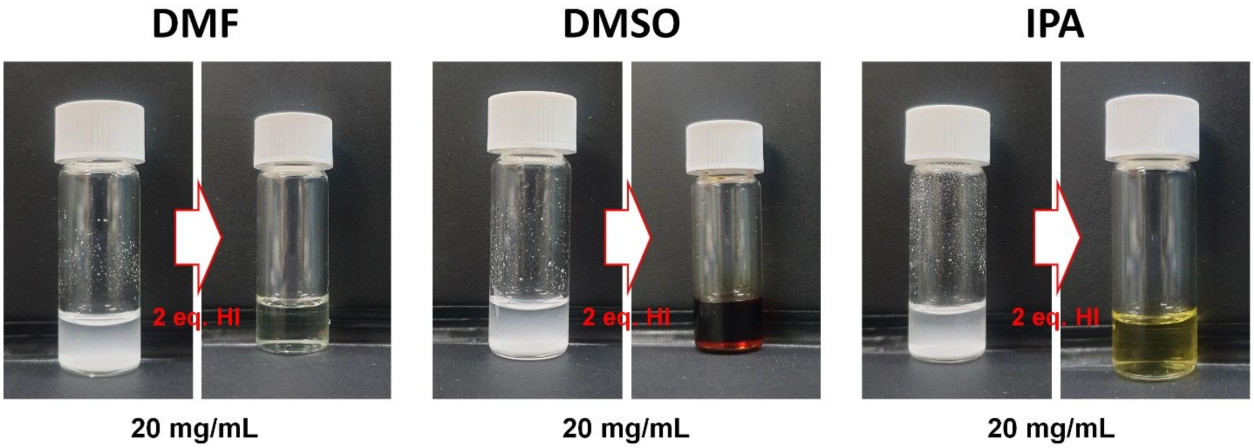

Figure S1. Solubility of creatine in DMF, DMSO and IPA.

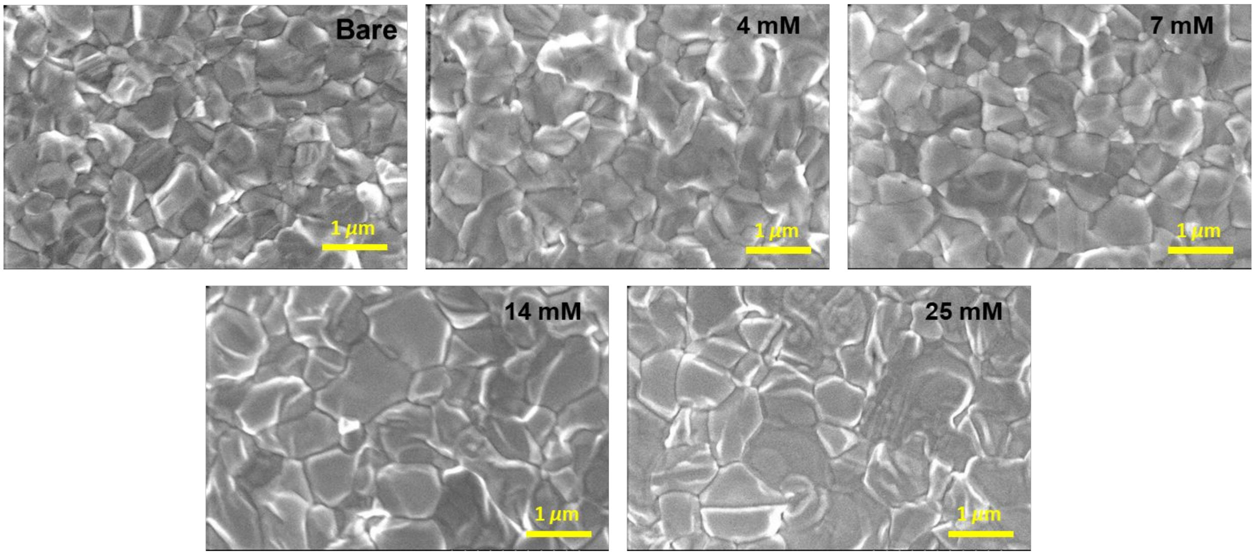

Figure S2. Top SEM images of perovskite layer with different concentration of CRI additives

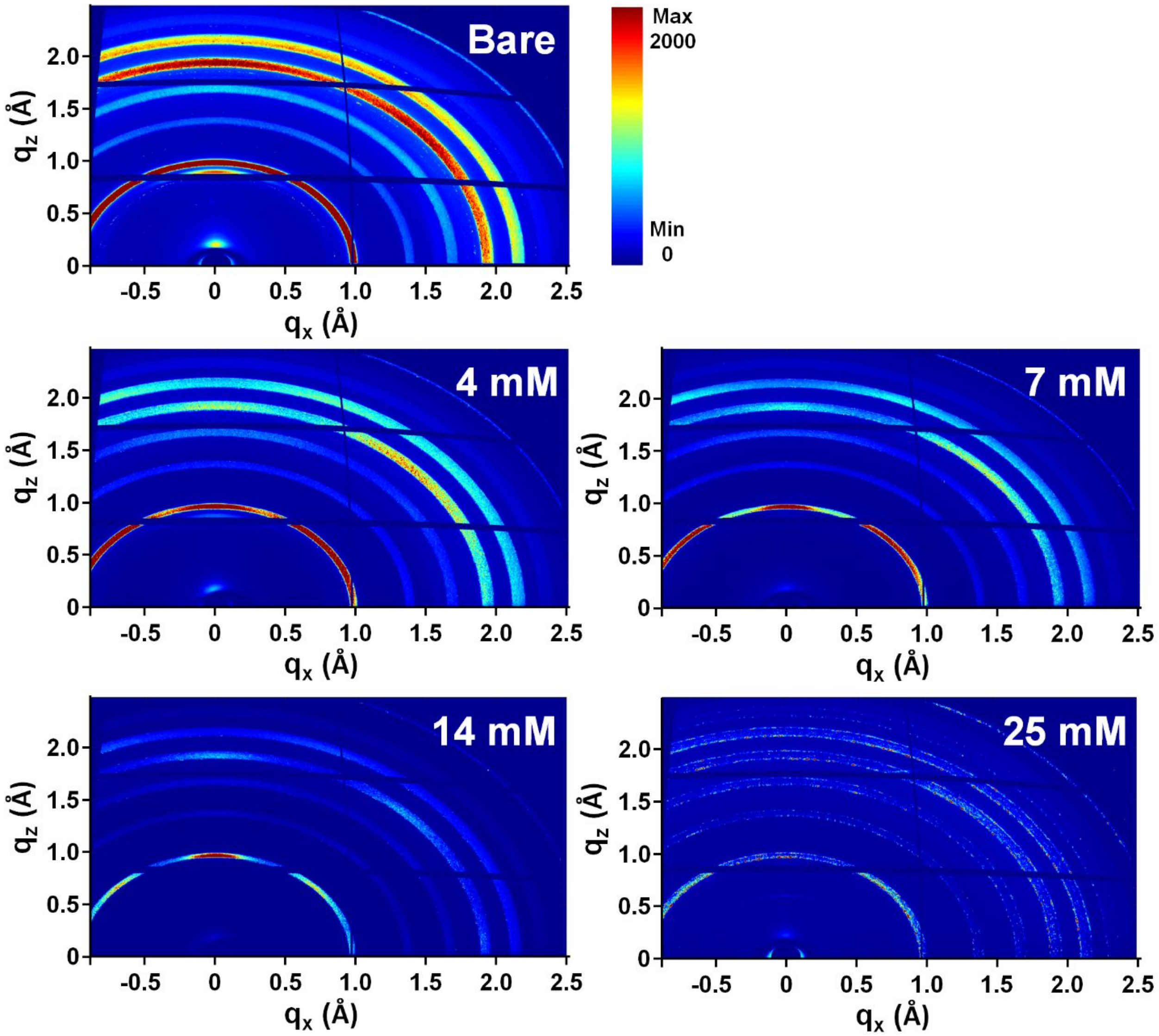

Figure S3. GIWAXS of perovskite layer with different concentration of CRI additives

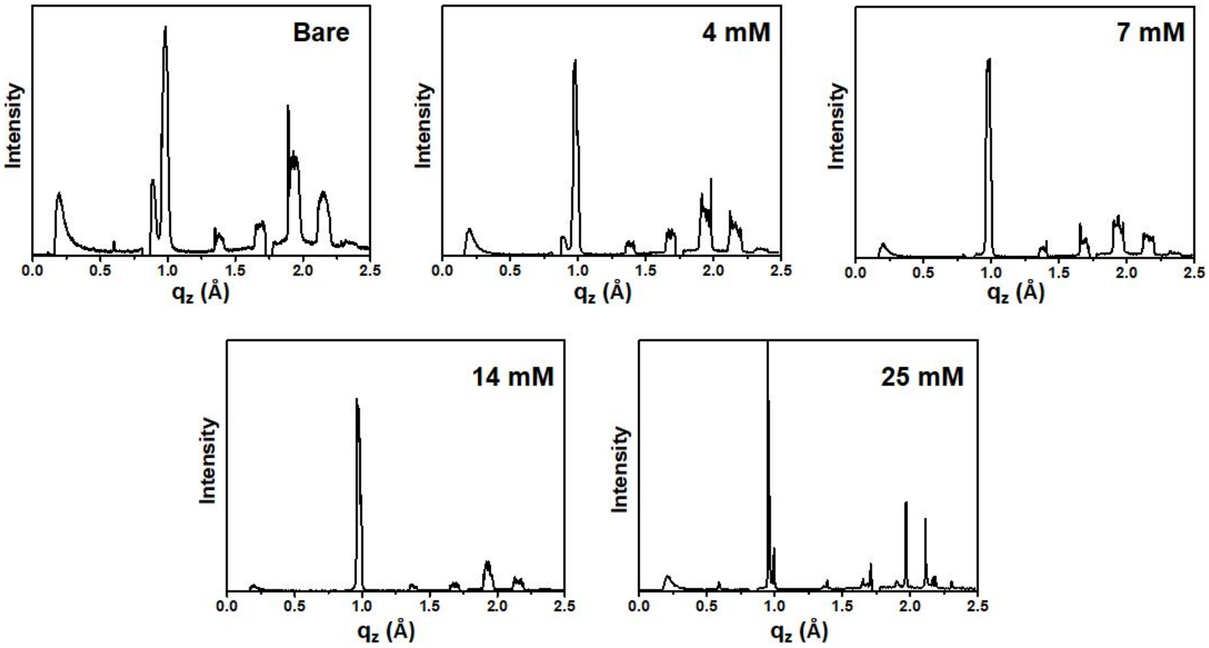

Figure S4. Radial cuts of perovskite layer with CRI additives.

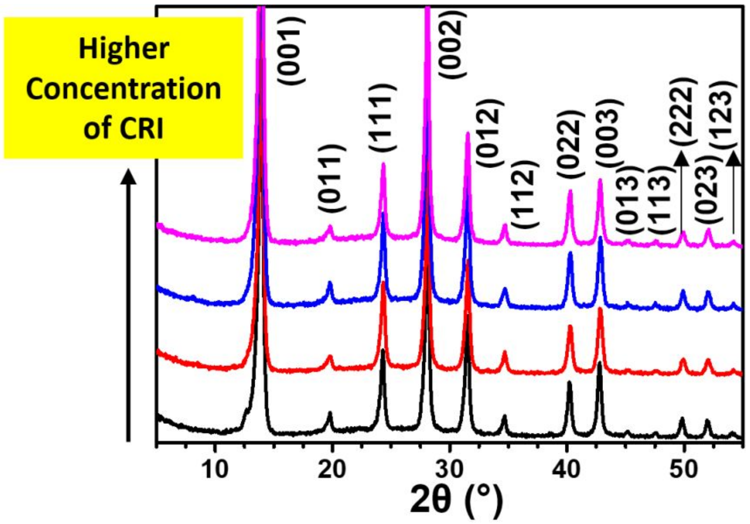

Figure S5. XRD of perovskite layer with different concentration of CRI additives

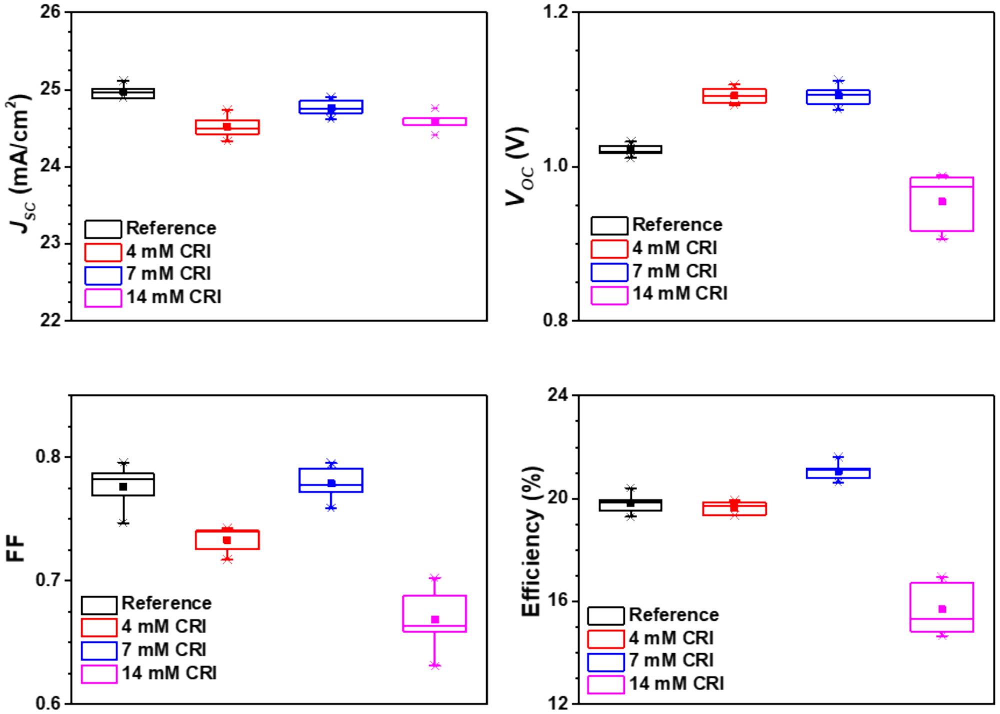

Figure S6. Histograms of perovskite solar cells with different concentration of CRI additives

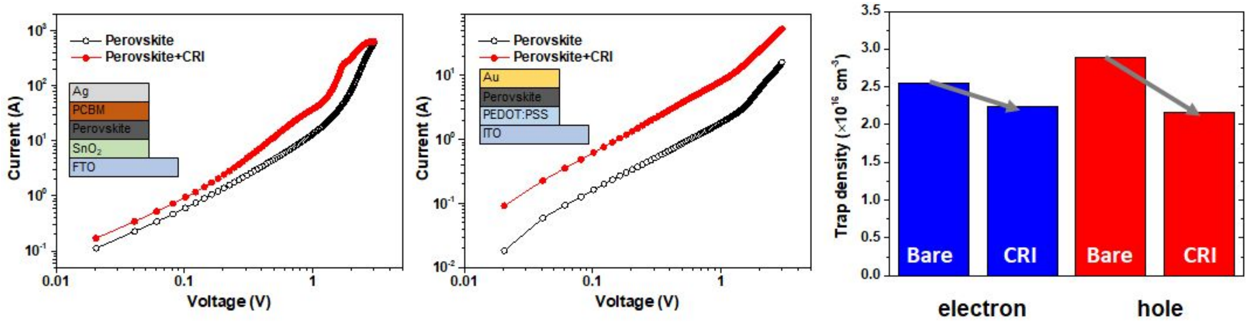

Figure S7. Current-voltage curves of SCLC devices and defect densities

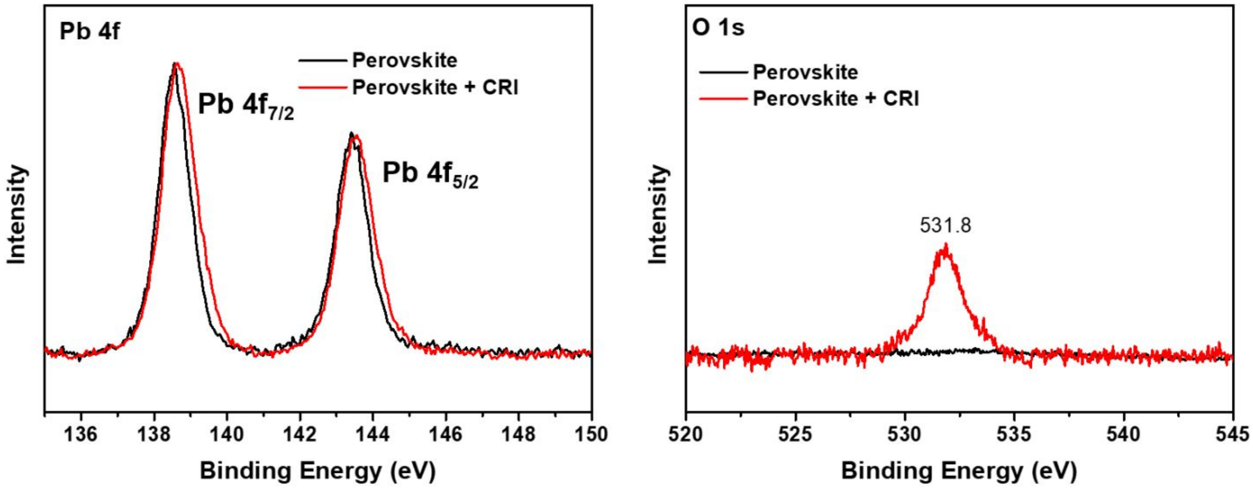

Figure S8. XPS of Pb 4f and O 1s of perovskite layer with and without CRI additives

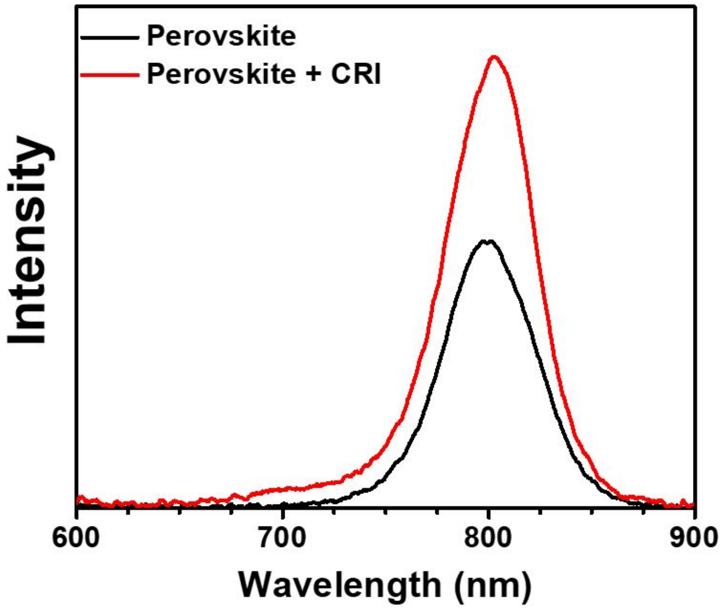

Figure S9. Steady state PL of perovskite layer with and without CRI additives prepared on bare glass.

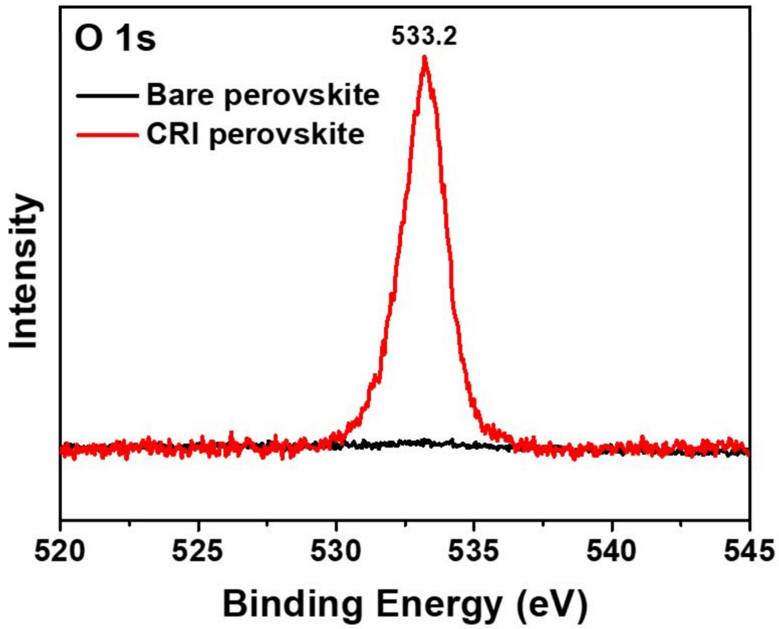

Figure S10. XPS of O1s of perovskite with CRI passivation layer

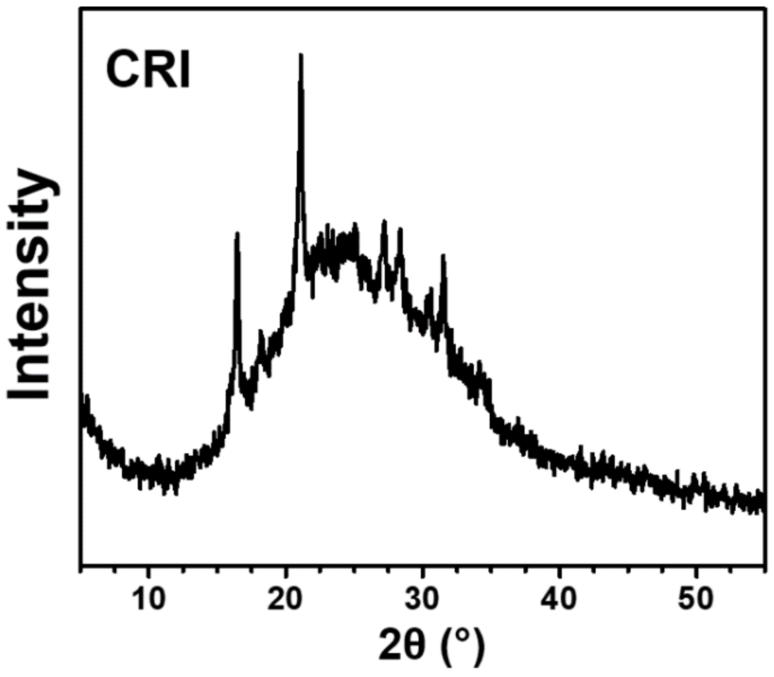

Figure S11. XRD of CRI

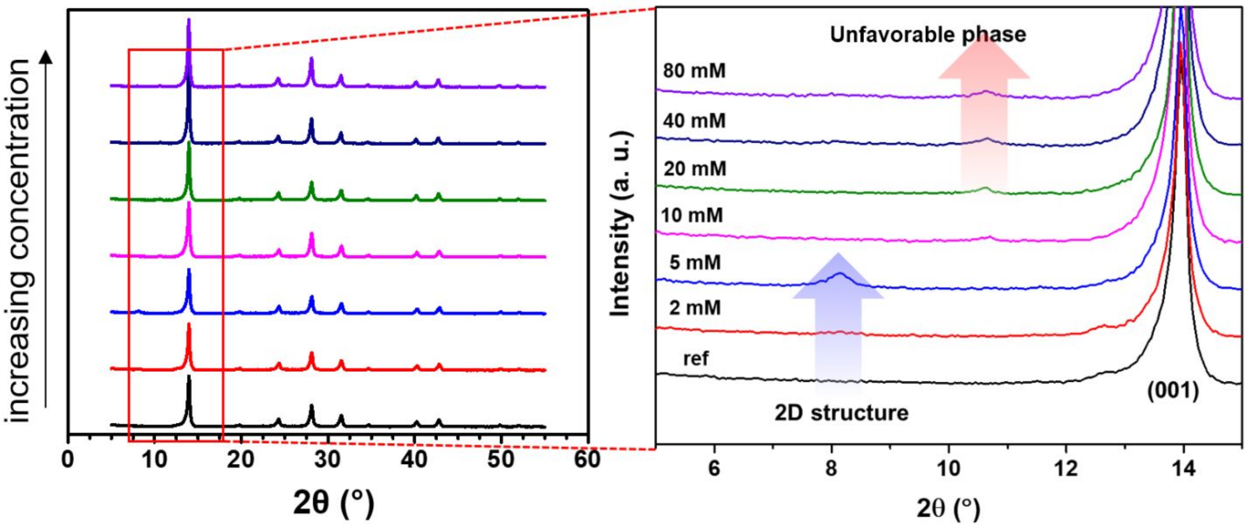

Figure S12. XRD of perovskite layer with different concentration CRI passivation layer

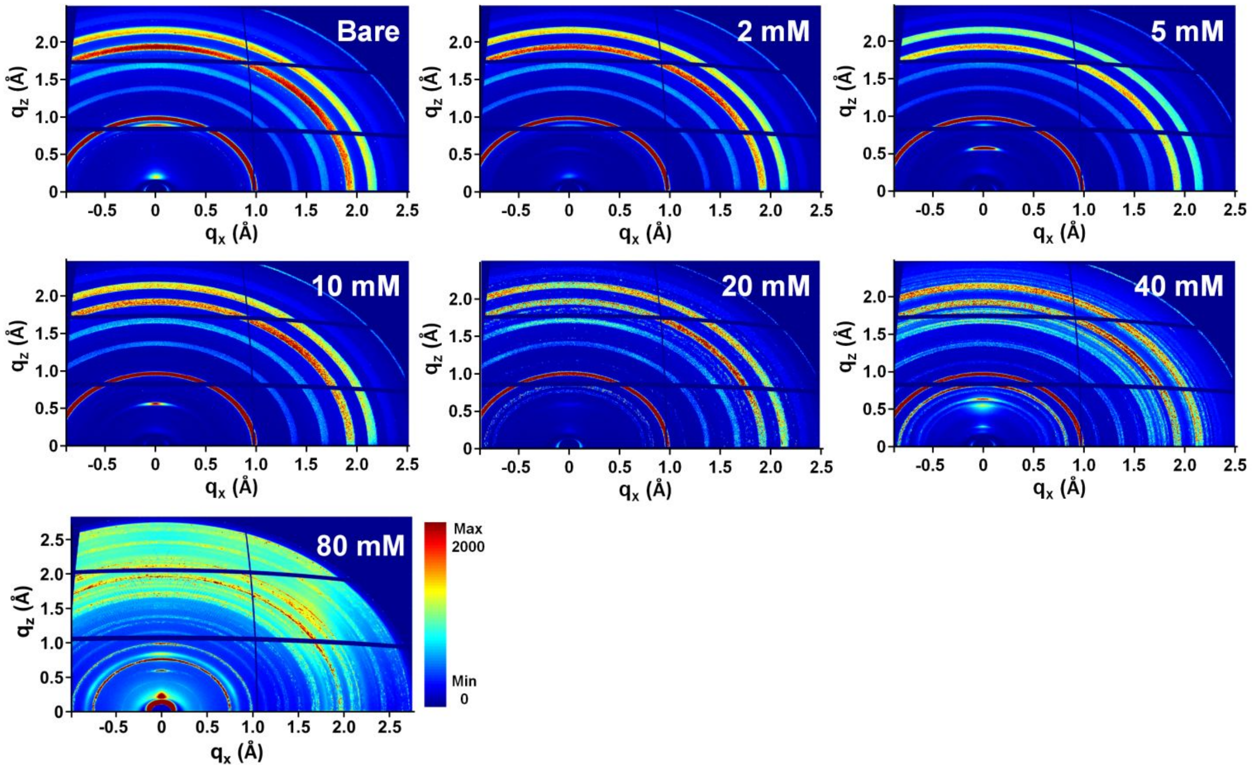

Figure S13. GIWAXS of perovskite layer with different concentration CRI passivation layer

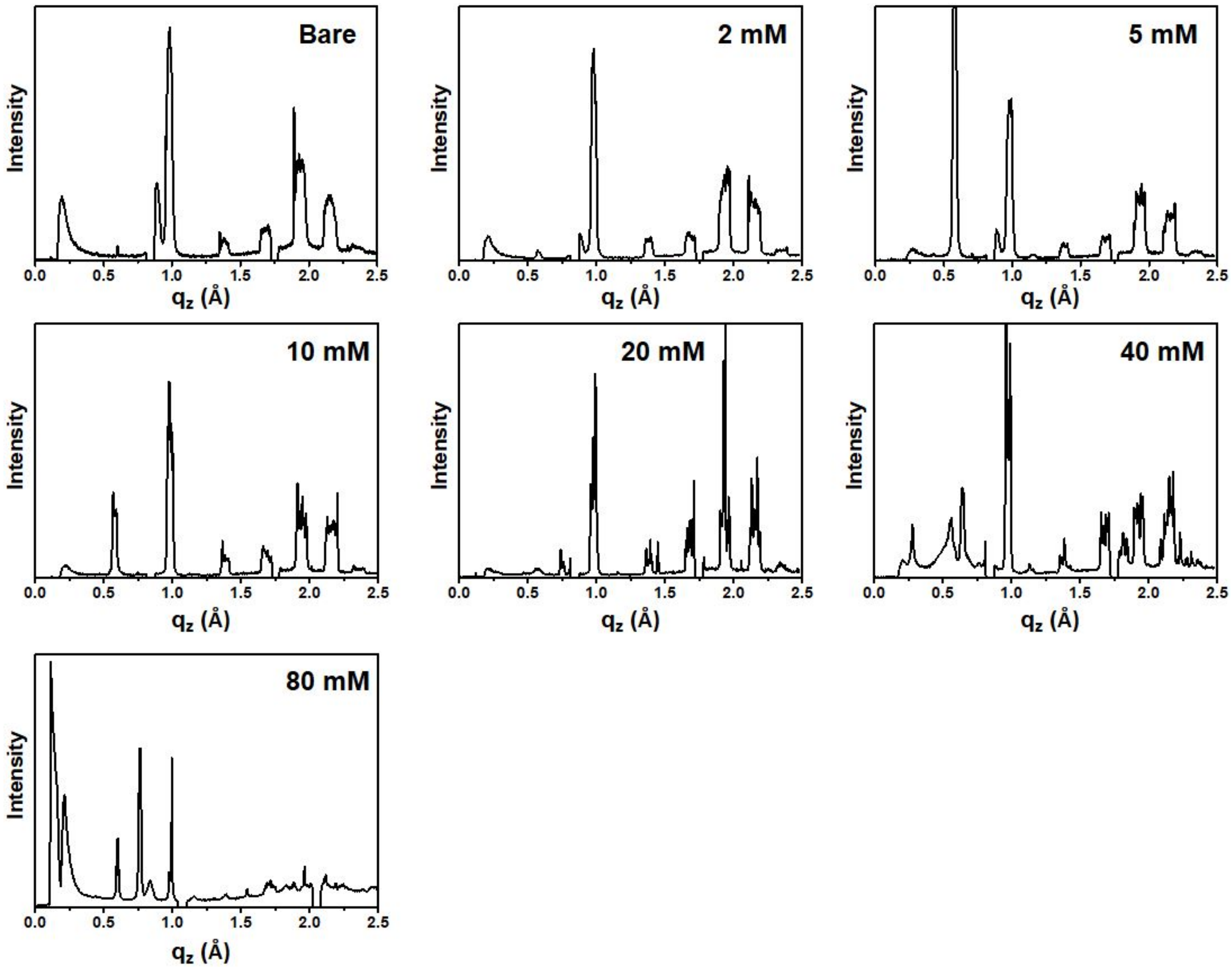

Figure S14. Radial cuts of perovskite layer with CRI overlayer.

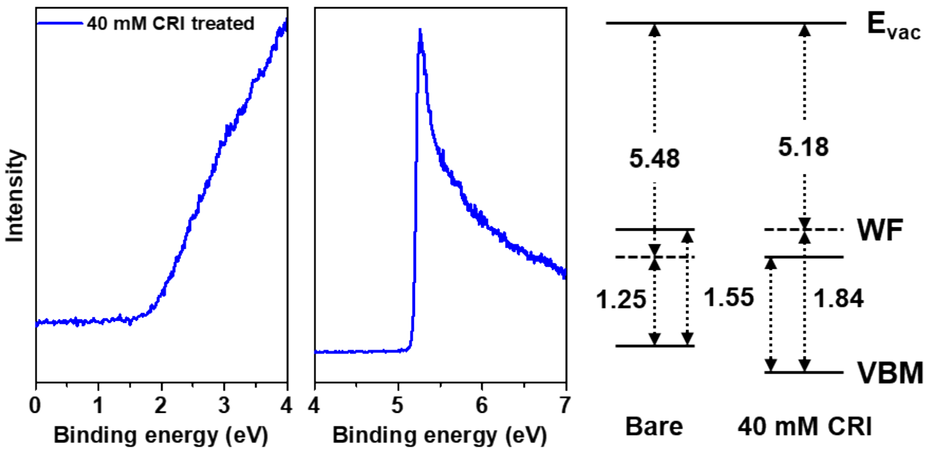

Figure S15. UPS of perovskite layer with 40 mM CRI passivation layer

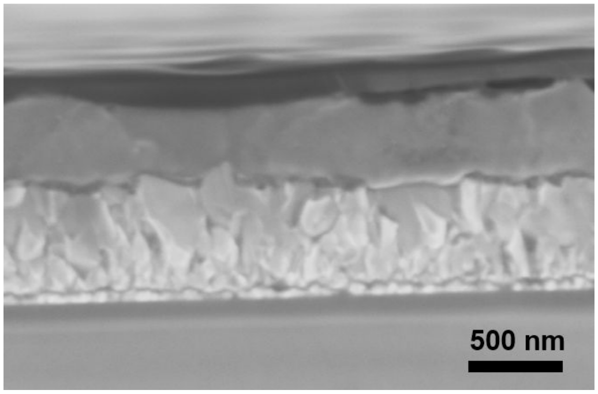

Figure S16. Cross sectional SEM image of planar perovskite solar cell

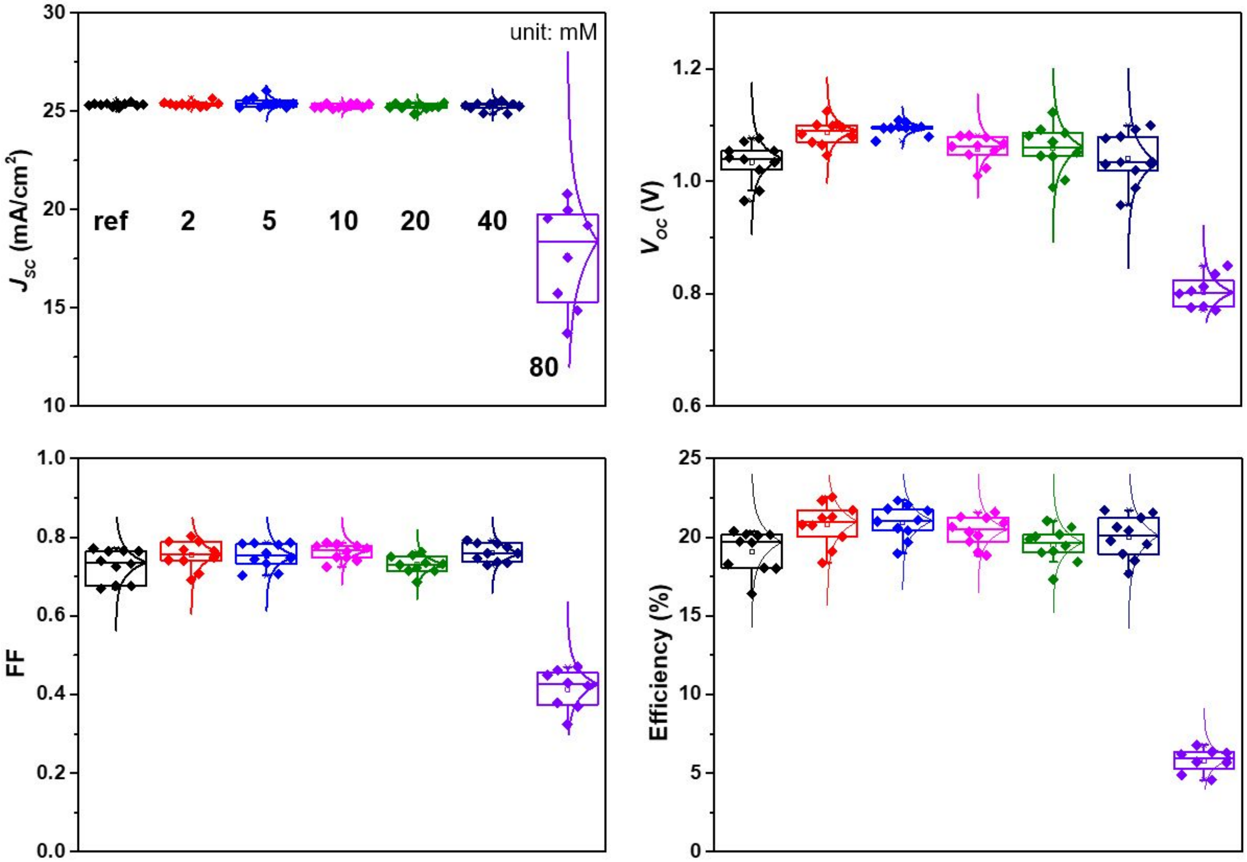

Figure S17. Box charts of devices using different concentration CRI passivation layer

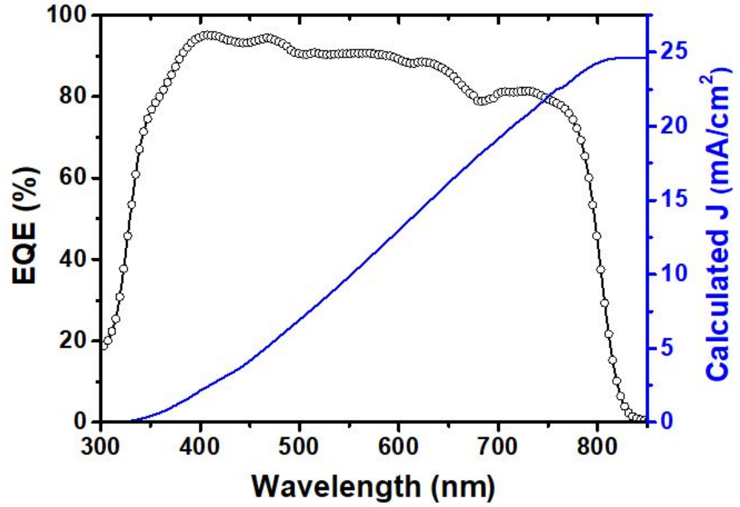

Figure S18. EQE of champion device

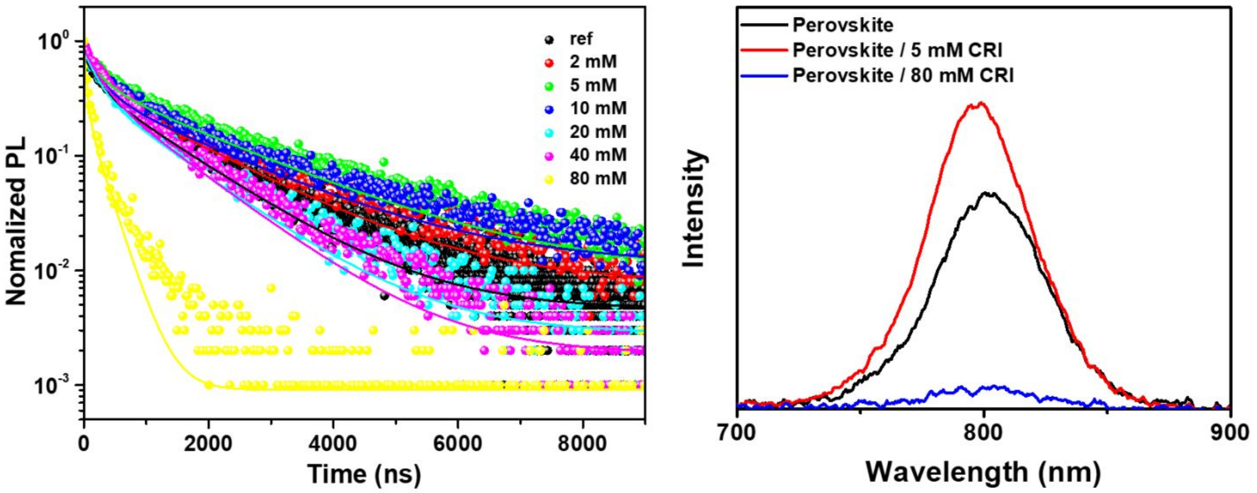

Figure S19. TRPL and steady state PL of perovskite layer with different concentration CRI passivation layer

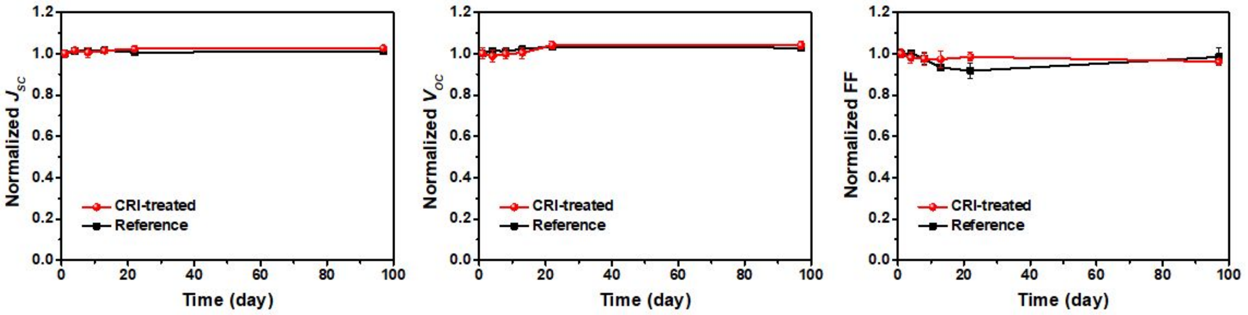

Figure S20. Shelf life stability tests: other parameters

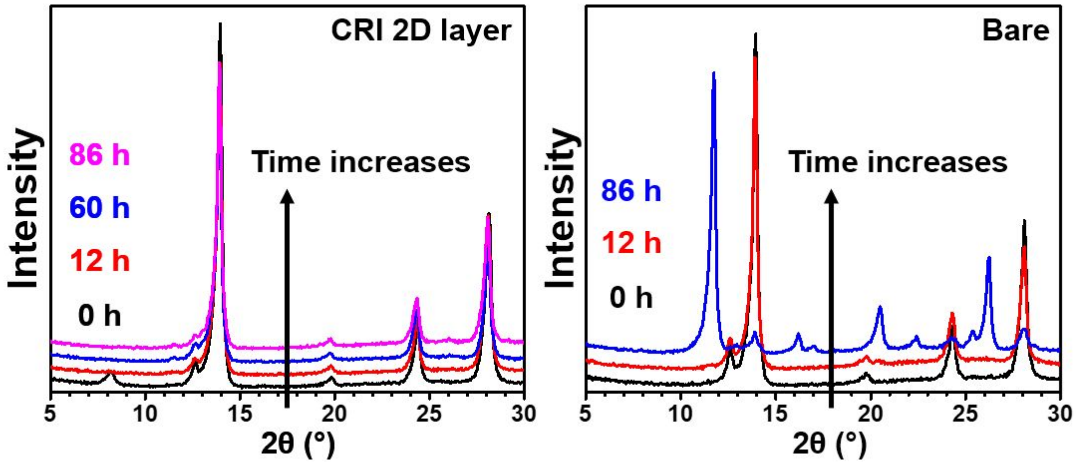

Figure S21. XRD changes with time at 50 %RH, RT.

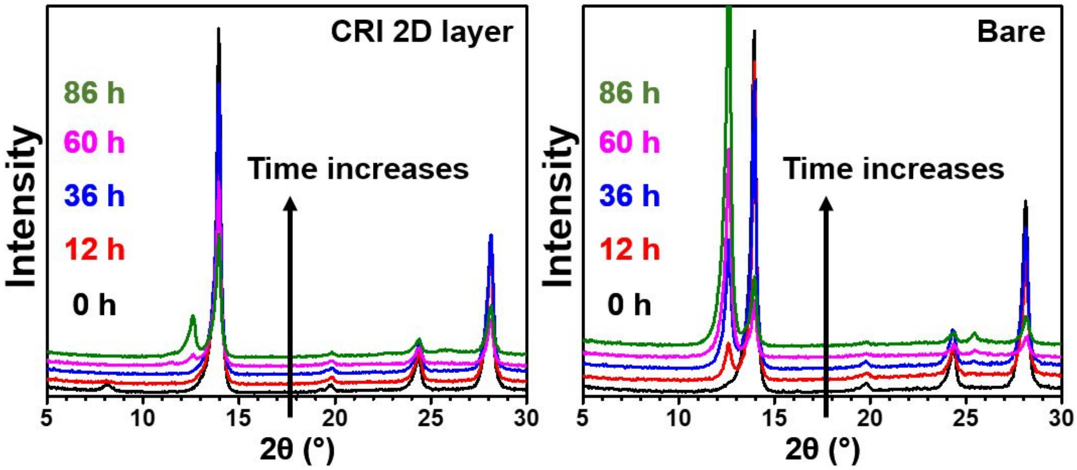

Figure S22. XRD changes with time at 50 %RH, 85 °C.

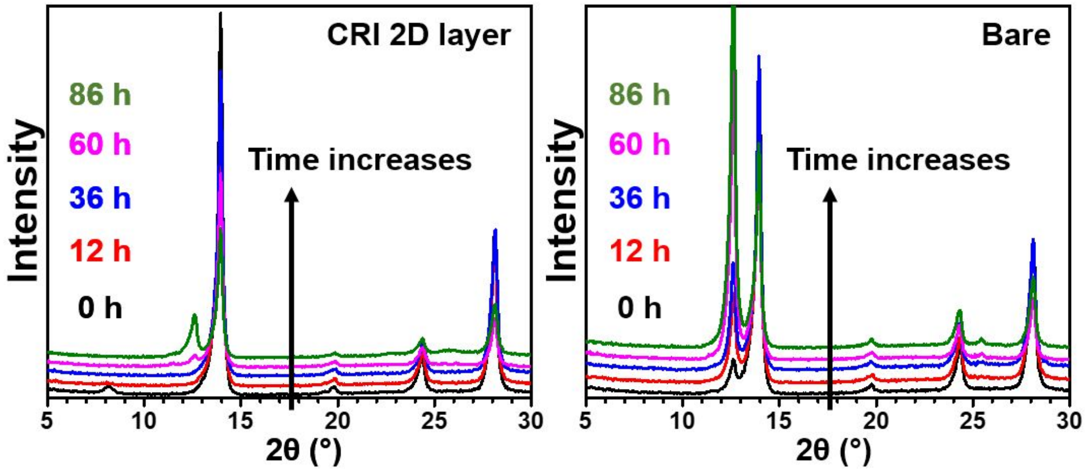

Figure S23. XRD changes with time at >80 %RH, 85 °C.

Table S1. Photovoltaic parameters of PSC employing CRI additives.

5 devices	Jsc (mA/cm^2)	Voc (V)	FF	Efficiency (%)
Reference	24.97±0.09	1.02±0.01	0.78±0.02	19.80±0.43
4 mM	24.52±0.16	1.09±0.01	0.73±0.01	19.64±0.28
7 mM	24.77±0.12	1.09±0.01	0.78±0.01	21.06±0.39
14 mM	24.58±0.13	0.95±0.04	0.67±0.03	15.69±1.07

Table S2. Photovoltaic parameters of PSC employing CRI passivation layer.

10 devices	Jsc (mA/cm^2)	Voc (V)	FF	Efficiency (%)
Reference	25.33±0.09	1.03±0.04	0.73±0.04	19.09±1.33
2 mM	25.37±0.12	1.09±0.02	0.75±0.04	20.81±1.34
5 mM	25.44±0.26	1.09±0.01	0.75±0.03	20.96±1.07
10 mM	25.26±0.11	1.06±0.02	0.76±0.02	20.37±0.94
20 mM	25.23±0.18	1.06±0.04	0.73±0.02	19.51±1.10
40 mM	25.25±0.21	1.04±0.05	0.76±0.02	20.00±1.35
80 mM	17.67±2.62	0.80±0.03	0.41±0.05	5.81±0.75
